# Optimization of 3D Printing Technology for Fabrication of Dental Crown Prototype Using Plastic Powder and Zirconia Materials

**DOI:** 10.3390/ma15238618

**Published:** 2022-12-02

**Authors:** Chonlada Bennett, Phanumas Sojithamporn, Warinthorn Thanakulwattana, Wassanai Wattanutchariya, Komgrit Leksakul, Wasawat Nakkiew, Kittisak Jantanasakulwong, Pornchai Rachtanapun, Jonghwan Suhr, Choncharoen Sawangrat

**Affiliations:** 1 Agriculture and Bio Plasma Technology Centre (ABPlas), Science and Technology Park, Chiang Mai University, Chiang Mai 50100, Thailand; 2 Department of Industrial Engineering, Faculty of Engineering, Chiang Mai University, Chiang Mai 50200, Thailand; 3 Advanced Manufacturing and Management Technology Research Center, Chiang Mai University, Chiang Mai 50200, Thailand; 4 Department of Agro-Industry, Faculty of Agro-Industry, Chiang Mai University, Chiang Mai 50100, Thailand; 5 Cluster of Agro Bio-Circular-Green Industry (Agro BCG), Chiang Mai University, Chiang Mai 50100, Thailand; 6 School of Mechanical Engineering, Sungkyunkwan University 2066 Seobu-ro, Jangan-gu, Suwon-si, Gyeonggi-do 16419, Republic of Korea

**Keywords:** fused deposition modeling, optimization, PLA composite, PLA/ZrO_2_, polylactic acid, prototype, zirconium dioxide

## Abstract

This research was aimed at developing a dental prototype from 3D printing technology using a synthetic filament of polylactic acid (PLA) and zirconium dioxide (ZrO_2_) with glycerol and silane coupling agent as a binder. A face-centered central composite design was used to study the effects of the filament extrusion parameters and the 3D printing parameters. Tensile and compressive testing was conducted to determine the stress-strain relationship of the filaments. The yield strength, elongation percentage and Young’s modulus were also calculated. Results showed the melting temperature of 193 °C, ZrO_2_ ratio of 17 wt.% and 25 rpm screw speed contributed to the highest ultimate tensile strength of the synthetic filament. A Nozzle temperature of 210 °C and an infill density of 100% had the most effect on the ultimate compressive strength whilst the printing speed had no significant effects. Differential scanning calorimetry (DSC) was used to study the thermal properties and percentage of crystallinity of PLA filaments. The addition of glycerol and a silane coupling agent increased the tensile strength and filament size. The ZrO_2_ particles induced the crystallization of the PLA matrix. A higher crystallization was also obtained from the annealing treatment resulting in the greater thermal resistance performance of the dental crown prototype.

## 1. Introduction

The production of dental prosthetics requires a great deal of expertise and specificity due to the difficulties in acquiring the desired look and feel for the original teeth. The re-placement of dental crowns or dentures in restorative dentistry has been employing the use of other non-traditional materials rather than the conventional acrylic resin or porcelain prosthetics. Ceramic combinations of monolithic zirconia and high-performance polymers have been reported [1]. Presently, the manufacturing of dental parts is manual, where tooth models are cast and coated with enamel material such as zirconia or zirconium dioxide (ZrO_2_). Ceramic materials such as ZrO_2_ are often used as they have very good mechanical and optical properties, are biocompatible and behave properly under cyclic fatigue [2,3]. The process of hand-casting dental models requires a skilled technician and is time-consuming. The lack of precision and accuracy due to human error is disadvantageous. Therefore, 3D modelling can be used to design prosthesis for input into milling machines referred to as CAD/CAM systems [4]. The use of CAD/CAM technology was introduced into dentistry to overcome some challenges. Tooth restorations became easier, faster and more accurate, providing patients with same-day restorations from on-site milling machines [5].

Three-dimensional (3D) molding technology has played a crucial role in solving problems and improving the medical industry. Currently, the adoption of 3D molding technology has many limitations in terms of cost, time and raw material [6]. The fabrication of dentures uses plastic, metal or ceramic materials [7]. Ceramic materials are often considered to be the main component in 3D printing due to their durability at high temperatures and abrasion resistance. It is inert to chemical reactions and resistant to microbial adhesion. Ceramic materials such as zinc or zirconium oxide are usually mixed with plastic pellets and molded into a desired shape. The dental mold undergoes a sintering process to change the crystal structure of the dentures and decompose the plastic resin so that the physical and mechanical properties of the dentures are consistent with dental applications [8]. However, the bonding forces between the ceramic and other dental materials may weaken over time [2].

Denture fabrication using 3D printing technology is reported to reduce the cost of production and decrease process time [9]. 3D printing is the process of forming workpieces by adding materials one layer at a time. These include industrial techniques known as rapid prototyping (RP) and rapid tooling (RT), where the visualization of the design uses computer-aided design (CAD) software [10]. The classification of 3D printing technology according to the ASTM standards includes material extrusion, powder bed fusion, vat photopolymerization, material jetting, binder jetting, sheet lamination and directed energy deposition [11]. Contour grafting, selective laser sintering, stereolithography, inkjet printing, laminated object manufacturing, electron beam welding and laser engineered net shaping are 3D printing technologies for the fabrication of synthetic materials. However, selective laser sintering (SLS), thermal inkjet printing (TIJ) and fused deposition modeling (FDM) are the only 3D printing technology used in the medical industry [12].

FDM is widely used in various industries for the fabrication of polymer composites because it is non-toxic and simpler than other printing techniques [13]. FDM can be used for all types of workpieces and have features that make it suitable for blending materials, thereby reducing the production of defects. In FDM, the plastic is melted into a liquid and injected through a nozzle. The 3D printer draws a line of the injected liquid material into a shape along the axis plane. One layer is completed at a time and continued to print for hundreds or thousands of subsequent layers until a desirable object is printed. Thermoplastics such as polylactic acid (PLA), polyethylene (PE) and acrylonitrile-butadiene-styrene (ABS) are often used due to their low melting point and can be remelted after cooling. PLA is a common thermoplastic that is biodegradable, biocompatible and environmentally friendly [14]. PLA with natural fiber as reinforcement has shown to increase the modulus and tensile strength [15].

The development of 3D modeling methods is constantly evolving, and the resultants of workpieces have greater precision, complexity and high accuracy [16]. The application of 3D printing is widely promoted in a range of industries such as engineering, design, aerospace and large-scale industrial productions. In recent years, researchers have utilized the application of 3D technology on a smaller and more complex level allowing the medical industry to develop alternative treatment options, including tissue organ production, prosthetic organs, neurosurgery, skull parts, artificial dentures, prosthetic legs and arms etc. [17]. Moreover, 3D printing technology has been incorporated into dentistry in the past decade but does not include ceramics and is limited to polymers [18].

In view of this, the aim of the research was focused on developing a prototype extruder for combining ZrO_2_ and polylactic acid (PLA). Furthermore, the improvement of 3D modeling for the fabrication of denture prototypes was investigated using response surface methodology (RSM). The current study aimed to develop an efficient method for denture production by adjusting and controlling the bioplastic (PLA) and ceramic material (ZrO_2_) mixture suitable for 3D printing. The parameters for filament extrusion were studied to obtain the most compatible filament composition. The extrusion of materials will enable the production of unique parts and increase the stability of the filament line during the manufacturing. Additionally, the optimum FDM 3D printer parameters for the fabrication of the denture prototype will be investigated. The FDM technology will facilitate the production of a dental crown prototype with complex structural shapes along with high resolution and precision.

## 2. Materials and Methods

### 2.1. Chemicals

Polylactic acid, glycerol, 3-glycidoxypropyltrimethoxy silane and zirconium dioxide were purchased from Sigma-Aldrich, St. Louis, MO, USA. The analytical grade reagent such as acetic acid, distilled water and ethanol were purchased from RCI Labscan limited, Bangkok, Thailand.

### 2.2. Filament Extrusion from PLA and ZrO_2_ Resins

PLA and 10 wt.% ZrO_2_ (1 μm) were blended in a ball mill with glycerol or silane to obtain four different types of filament composition, including PLA/ZrO_2_, PLA/ZrO_2_/glycerol, PLA/ZrO_2_/silane and PLA/ZrO_2_/glycerol/silane. The moisture contents were removed, and the composite materials were extruded using a single screw extruder (Wellzoom, Shenzhen, Guangdong, China) with a melting temperature of 190 °C and screw speed of 20 rpm. Prior to ball milling, a concentration of 3% *v*/*v* silane was added to a solution containing 95% *v*/*v* ethanol and 5% *v*/*v* distilled water, adjusted to pH 4–4.5 with acetic acid and left to stir for 15 min. ZrO_2_ was soaked for 8 h and washed with distilled water. The moisture content was removed using an oven at 120 °C before extrusion with PLA [19]. The tensile strength for each type of filament was compared where 200 mm of filament was cut and pulled at a rate of 10 mm min^–1^ [20]. The filament composition with the highest tensile strength was chosen for further investigation to determine the most suitable factors for filament extrusion via design of experiment.

### 2.3. Design of Experiment for Filament Extrusion

Filament extrusion factors such as melting temperature, PLA/ZrO_2_ ratio and screw speed were optimized using a face-centered central composite design (CCF) as shown in Table 1. Design of experiment used Minitab version 17.0 (Minitab LCC, State College, PA, USA). Multiple regression analysis was used to analyze the relationship between the factors and the ultimate tensile strength. The uniformity of the synthetic filament size was measured randomly (20 points) using an 8-inch vernier digital caliper (BEC, Jiangsu China).

### 2.4. Design of Experiment for 3D Printing

The FDM 3D printer (Pro2 Plus, Raise3D, Irvine, CA, USA) parameters such as nozzle temperature, infill density and printing speed were optimized using CCF as shown in Table 2. PLA/ZrO_2_ were molded into a cylinder with a diameter and height of 10 mm [21]. The parameters for the 3D printer were programmed with ideaMaker version 3.3.0 (Raise3D). The 3D printer bed was heated to 60 °C. Multiple regression analysis was used to determine the optimal condition using the factors and the ultimate compressive strength. The compressive strength was tested by applying a pressure of 10 kN load cell at a rate of 0.5 mm min^−1^ using a Universal Testing Machine (model 5566, Instron, Norwood, MA, USA). The dental crown prototype was printed with a nozzle temperature of 210 °C, infill density of 100% and print speed of 60 mm s^−1^. The 3D model of the dental crown prototype was obtained from TurboSquid website (TurboSquid, New Orleans, LA, USA) [22].

### 2.5. Scanning Electron Microscopy

The surface microstructure of the PLA/ZrO_2_ filament prepared under four different conditions were analyzed using scanning electron microscopy (SEM). The distribution of ZrO_2_ particles on PLA polymer surface after molding was observed at 10.0 kV using a secondary electron detector.

### 2.6. Differential Scanning Calorimetry

The physical property and percent crystallinity of the PLA/ZrO_2_ filaments prepared under four different conditions were tested using a differential scanning calorimeter (DSC) where the melting temperature was obtained and used to determine the nozzle temperature of the FDM 3D printing as described in Section 2.5. DSC of the composites was conducted in the temperature range of 25 to 200 °C, heating rate 10 K min^−1^. The percentage of crystallization (%*χ_c_*) was calculated using the following Equation (1); where Δ*H_m_*, Δ*H_c_* and Δ*H_m°_* represents the enthalpy of melting, the enthalpy of cold crystallization and the enthalpy of 100% PLA (93.7 J g^−1^), respectively [23].

(1) $$\chi_{c}\left(\%\right)=\frac{\Delta H_{m}-\Delta H_{c}}{\Delta H_{m}{}^{\circ}}\times 100$$

### 2.7. Annealing Treatment

The mechanical properties of the PLA/ZrO_2_ filaments were studied by comparing the compressive strengths of the annealed and unannealed composites printed with a nozzle temperature of 210 °C, infill density of 80% and print speed of 100 mm s^−1^. The composites were annealed at 80 °C for 30 min using an incubator (BPG-9070A, Ponpe, Pathum Thani, Thailand) [24]. The yield strength, elongation percentage and Young’s modulus were also calculated following equations in Appendix A, Equations (A1)−(A5).

## 3. Results

### 3.1. Comparison of PLA and ZrO_2_ Filament Composition

**Figure 1:**
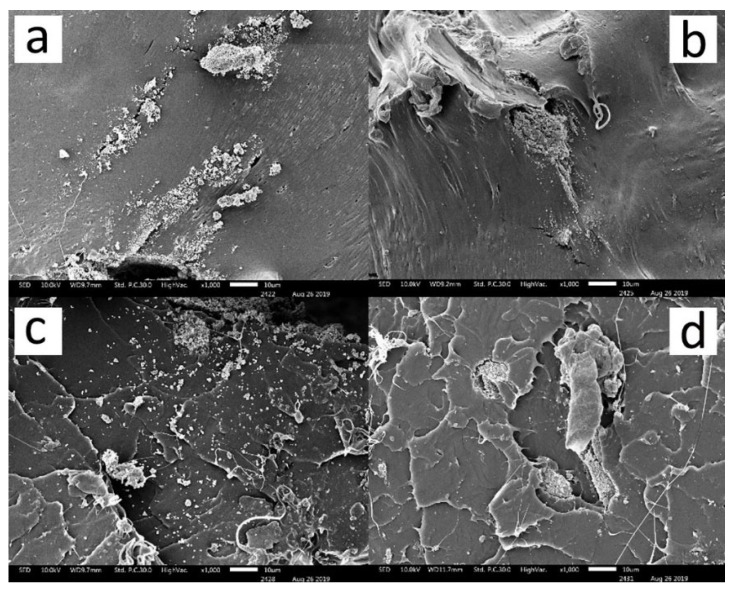
ZrO_2_ particle dispersion characteristic on (**a**) PLA/ZrO_2_ (**b**) PLA/ZrO_2_/Glycerol (**c**) PLA/ZrO_2_/Silane and (**d**) PLA/ZrO_2_/Glycerol/Silane polymer substrate at 1000 × magnification.

Filament compositions of PLA/ZrO_2_, PLA/ZrO_2_/glycerol, PLA/ZrO_2_/silane and PLA/ZrO_2_/glycerol/silane were investigated by melt-mixing PLA with ZrO_2_ (1 µm of particle size) at a ratio of 10 wt.%. Silane and glycerol coupling agents were added and the mechanical properties of the filaments were tested. Results for the tensile strength and physical characteristics of the plastic filaments are shown in Table 3. Both ultimate tensile strength and yield strength were measured to be greater in the order of PLA/ZrO_2_/glycerol/silane, PLA and PLA/ZrO_2_/silane. The stress-strain relationship explained by Young’s modulus showed no statistical difference between the different filament compositions. PLA/ZrO_2_ filaments gave the lowest ultimate tensile strength, elongation percentage and yield strength with a significant difference due to incompatibility between PLA and ZrO_2_. This was consistent with the SEM result which characterized the distribution of the zirconia particles on the PLA polymer substrate (Figure 1). Filament composition of PLA/ZrO_2_ had a rough and uneven surface due to the nonuniform distribution of ZrO_2_ powder (Figure 1a). The addition of glycerol improved the ultimate strength and elongation percentage for the PLA/ZrO_2_/glycerol filament composition. The use of the silane coupling agent enhanced the compatibility of the PLA and ZrO_2_ resin which increased the ultimate tensile strength by 59%. The chemical properties of the synthetic filaments were not affected by the addition of the glycerol and silane coupling agent. This was displayed in the SEM examination (Figure 1b–d) where the addition of glycerol and the silane coupling agent increased the dispersion of ZrO_2_ on the polymer surface and less ZrO_2_ powder agglomerations on the PLA substrate were observed. As a result, the PLA/ZrO_2_/glycerol/silane filament composition obtained the highest values for the ultimate tensile strength, elongation, Young’s modulus and yield strength. This filament composition was selected for further investigation to find the most appropriate factors for filament extrusion using design of experiment.

### 3.2. Optimization of Filament Extrusion

**Figure 2:**
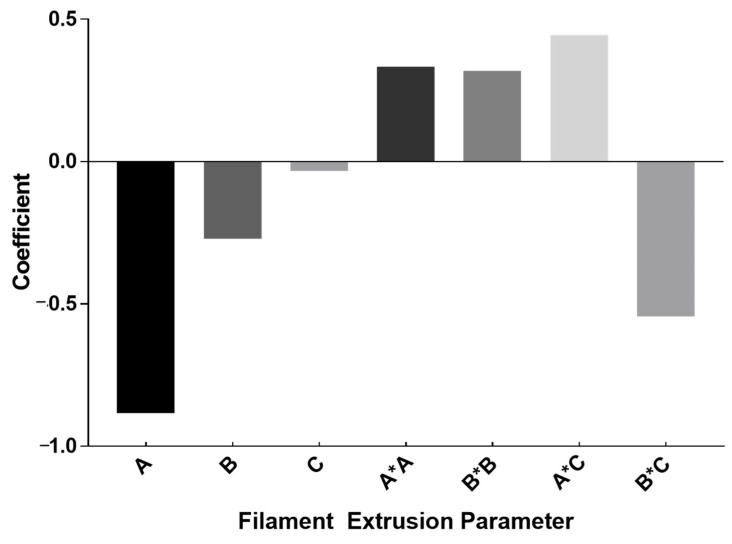
Coefficients for filament extrusion optimization model showing significant factors; including melting temperature (A), ZrO_2_ ratio (B), screw speed (C), quadratic effect of melting temperature (A*A), quadratic effect of ZrO_2_ ratio (B*B), interaction effect of melting temperature and screw speed (A*C) and interaction effect of ZrO_2_ ratio and screw speed (B*C).

The PLA/ZrO_2_/glycerol/silane filament extrusion parameters were optimized including melting temperature, ZrO_2_ ratio, and screw speed. Each factor was assigned 2 levels, with a total of 20 experiments. Results for the ultimate tensile strength were analyzed using the quadratic regression which obtained a set of coefficients, graphically depicted in Figure 2. The graph represents statistically significant results at *p* < 0.05. The relationship between the experimental factors and response (ultimate tensile strength) obtained coefficients which were used to describe the positive and negative effects of each parameter. The model summary obtained an R^2^ of 93.04% and R^2^-adjusted of 88.98%. The regression equation for significant model terms is represented by the following equation:

(2) Ultimate tensile strength=18.462−0.8832 A−0.2717 B−0.3286 C+0.3338 A∗A+0.3179 B∗B+0.445 A∗C−0.545 B∗C

**Figure 3:**
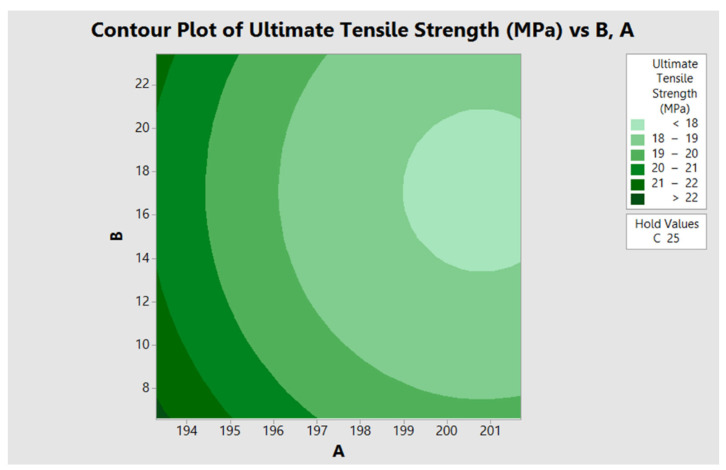
Contour plot of the ultimate tensile strength versus filament extrusion parameters; melting temperature (A) and ZrO_2_ wt.% (B).

The melting temperature (A), ZrO_2_ ratio (B) and screw speed (C) had a negative linear effect on the tensile strength of filaments, with significance at *p* < 0.05. This was evident when the amount of ZrO_2_ ratio was increased. The ZrO_2_ particles were agglomerated into clumps, resulting in the uneven extrusion of the filament. ZrO_2_ ratio of 17 wt.% gave the highest tensile strength and consistent filament diameter of 0.75 ± 0.05 across the filament line. The melting temperature and ZrO_2_ ratio showed significant quadratic effects suggesting a curvature in the result. A positive interaction effect was observed for the melting temperature and screw speed. However, a negative interaction effect was determined for the ZrO_2_ ratio and screw speed, with significance at α = 0.05. Regardless of the ZrO_2_ ratio and screw speed, the ductility of the filament was mostly influenced by the melting temperature. It was noted that the filament extrusion parameter with the highest melting temperature, ZrO_2_ ratio and screw speed had low ductility or a low elongation percentage (11.73 ± 1.06%). However, the filament with the highest elongation percentage (19.63 ± 1.90%) was obtained from the experiment with the lowest melting temperature, highest ZrO_2_ ratio and highest screw speed. It was concluded that both low and high ZrO_2_ ratio and screw speed corresponded to low ductility with statistical difference. It was evidenced in the contour plot (Figure 3) that the high melting temperature corresponded to low tensile strength. The response optimizer was applied to obtain the optimal condition for filament extrusion using PLA and ZrO_2_. The ultimate tensile strength was targeted at 35 MPa to compromise with the 3D printing technology parameters. The optimized condition for the maximum compressive strength was found with the melting temperature of 193 °C, ZrO_2_ ratio of 17 wt.% and screw rotation speed of 25 rpm, which had a desirability of 1.0000.

### 3.3. Optimization of 3D Printing via FDM

The FDM 3D printing parameters studied were nozzle temperature (A), infill density (B) and print speed (C). The model summary obtained an R^2^ of 85.82% and R^2^-adjusted of 73.06%. The interaction effect between two parameters, nozzle temperature and infill density, were found to be significant. The result from the optimization model is concluded by the following equation:

(3) Ultimate compressive strength=51.28+8.39 A+7.82 B−5.14 A∗B

**Figure 4:**
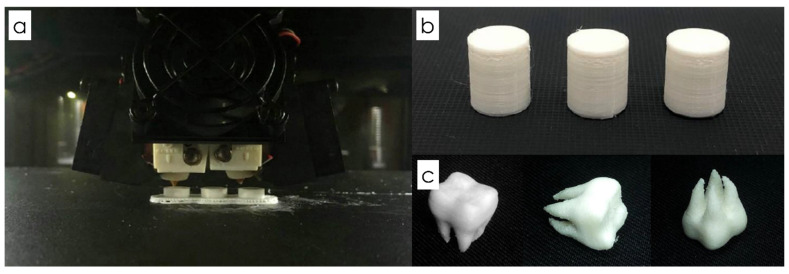
(**a**) FDM 3D printing technology in progress (**b**) FDM 3D printed cylinders and (**c**) dental crown prototype made from PLA synthetic resin and ZrO_2_.

The nozzle temperature and infill density had a positive linear effect on the ultimate compressive strength, whilst the print speed had no effects with statistical significance. The optimum condition for FDM 3D printing was calculated using the response optimizer where the targeted value for the result was the maximum compressive strength. It was found that in order to obtain the highest compressive strength, 62 ± 1.22 MPa, the nozzle temperature should be adjusted to 210 °C and the infill density at 100%, respectively. The optimized FDM experimental parameter for the highest compressive strength (62 ± 1.22 MPa) gave a composite desirability of 0.93342, indicating that the derived factor values were appropriate. From this, a dental prototype was formed using PLA and 17 wt.% ZrO_2_ resins and FDM 3D printing technology with a nozzle temperature of 210 °C, infill density at 100% and printing speed of 60 mm min^−1^. The filament composite demonstrated high heat resistance properties and was able to withstand compressive strength of 62MPa. An example of the dental crown prototype is shown in Figure 4. A dental prototype was formed using PLA and 17 wt.% ZrO_2_ resins via FDM 3D printing technology with a nozzle temperature of 210 °C, infill density at 100% and printing speed of 60 mm min^−1^.

### 3.4. Thermal and Mechanical Properties of Annealed Filaments

**Figure 5:**
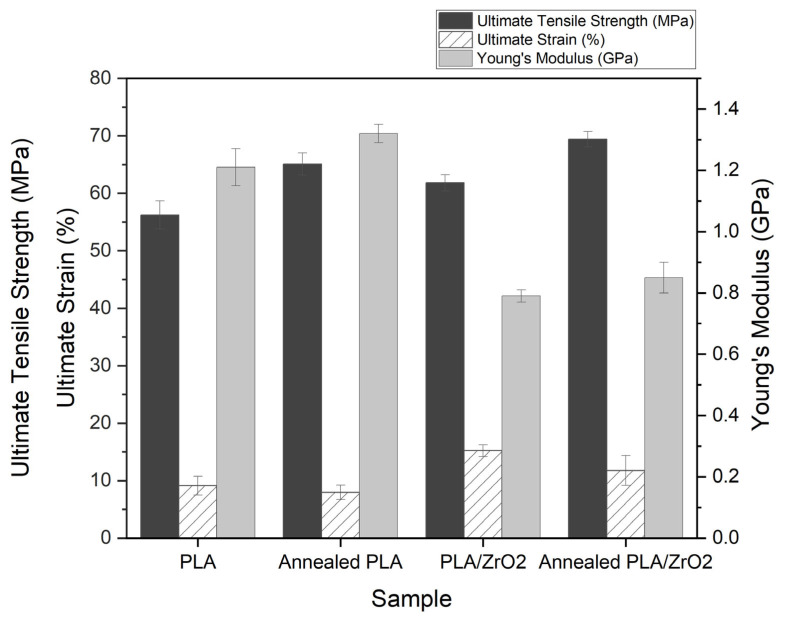
Thermal and mechanical properties of annealed PLA and PLA/ZrO_2_ composites.

The thermal properties of PLA and PLA/ZrO_2_ filaments were analyzed using DSC and showed the percentage of crystallinity to increase for annealed filaments in comparison to the unannealed filaments (Table 4). The annealed PLA/ZrO_2_ composite had the highest percentage of crystallinity (9.43 ± 1.65%) in comparison to the annealed PLA filament (7.47 ± 1.25%). The presence of ZrO_2_ particles induced the crystallinity of the PLA matrix by 2.20% which increased to 3.49% after annealing. The crystallinity of the PLA filament after annealing increased by 3.73%. The annealing process at 80 °C for 30 min accelerated the crystal formation of the PLA matrix. The thermal and mechanical properties of the annealed composite resulted in increased heat resistance and compressive strength as compared to the unannealed composites. The annealing process improved the compressive strength by 15.82 ± 1.01% for PLA and 12.24 ± 1.25% for PLA/ZrO_2_ composites (Figure 5). The annealing process accelerated the formation of PLA crystals with strong interaction in the PLA structure, which affected the compressive strength of the PLA/ZrO_2_ samples, respectively.

### 3.5. Comparison of PLA Synthetic Filament before and after Optimization Processes

In this study, the optimization of PLA filament compositions, filament extrusion and FDM 3D printing parameters were investigated. It was found that the optimized processing conditions improved the mechanical properties of the PLA synthetic filament with statistical difference. The PLA filament before the optimization gave the yield strength of 15.10 ± 1.66 MPa and an ultimate tensile strength of 22.68 ± 1.18 MPa. The PLA filament before the optimization used plastic resin without any additives such as zirconia, glycerol or silane coupling agent. It was observed that the PLA filament before the optimization was not able to withstand the tension from the load cell and the transition of the filament from the elastic to the plastic region was lower than that of the PLA filaments after the optimization and annealing processes. The optimization of PLA filaments included the composition of the synthetic material, the ratio of ZrO_2_, the temperature for extrusion as well as the FDM 3D printing parameters. The ultimate strength of the filament at the optimal condition and after annealing was increased to 54.52 ± 2.18 MPa and 69.41 ± 1.35 MPa, respectively.

This increase was believed to result from the chain arrangement, interfacial reaction and interaction of PLA/ZrO_2_ via silane during the annealing process. The elongation percentage of the filament before optimization was found at 14.55 ± 0.61% and increased to 18.51 ± 1.10% at the optimal condition. After the annealing process, the elongation of the filament was found at 11.76 ± 2.60%. This suggests that the annealing process increased the observed ductility of the filaments. Correspondingly, the Young’s modulus was found to increase for the PLA filament before and after the optimization and after annealing, respectively.

## 4. Discussion

The optimization of PLA/ZrO_2_ synthetic polymer was conducted for the fabrication of a dental crown prototype via 3D printing. The mixing of ZrO_2_ was reported to harden the PLA matrix and make the filament less flexible. The PLA/ZrO_2_ filament was brittle and fragile, especially in the areas where ZrO_2_ powders were agglomerated. The non-homogeneity between the polymer and the ceramic powders reduced the molding efficiency of the PLA/ZrO_2_ filaments. In view of this, glycerol was introduced to increase the dispersion of the zirconia particles on the polymer matrix. Glycerol is reported to contain multiple hydroxyl groups (−OH) which are soluble in both polar organic and inorganic compounds [25]. The addition of glycerol improved the mechanical properties of the plastic filaments by helping the even distribution of ZrO_2_ powder. Additionally, the silane coupling agent, 3-glycidoxypropyltrimethoxy silane, was studied due to the capability of the epoxy functional groups to form a reaction with the carboxyl functional groups of the PLA [26]. The epoxy groups of silane were indicated to form a reaction with -COOH end groups of PLA while the Si of the silane structure formed interactions with ZrO_2_ to increase interfacial adhesion between PLA and ZrO_2_. Favier and co-author [27] described the use of glycerol as a solution and catalyst, which was found to help disperse the metal particles to remain stable in solution. The binding of the silane coupling agent enhanced the affinity of the polymer and zirconia resin due to interfacial adhesion [28]. The selection of denture base material and resin is crucial to the denture longevity, as it is dependent on the surface characteristic and surface properties [29].

The investigation on optimization parameters for filament extrusion showed the melting temperature to have the most significant effect. However, filament extrusion parameters such as ZrO_2_ ratio and screw speed contributed to the uniformity and strength of the filament. The tensile strength of the filament was largely affected by the homogeneity of the mixture during extrusion. A recent study suggested that the homogeneity of the mixture during extrusion may have been insufficient due to the amount of oxide present which could change the viscosity of the filament [30]. It was suggested in the literature that ZrO_2_ particles were able to induce the PLA matrix to crystallize more effectively [24]. This is because ZrO_2_ particles acted as a nucleating agent which accelerated the formation of crystals in PLA and improved the thermal and mechanical properties of the PLA/ZrO_2_ samples.

In 3D printing, the nozzle temperature and infill density were known to be significant factors which affected the strength of the filament’s workpieces. The nozzle temperature is based on the melting point of materials which plays a vital role in 3D printing as it can affect layer adhesion whilst the infill density does the mechanical properties, stiffness and shape of the workpiece [31]. The nozzle temperature could affect the strength and stiffness of the filament as an increase in nozzle temperature could enhance the chain diffusion of the polymer [32]. In the literature, FDM workpieces with the maximum strength and Young’s modulus was achieved at 210 °C nozzle temperature and 100% infill density [33]. Tlegenov (2018) reported that PLA was more sensitive to the change in nozzle temperature as compared to ABS [34]. This is because additional force is required for the PLA with higher viscosity and brittleness to extrudate through the nozzle whilst ABS was less inclined to clog during extrusion. Furthermore, the rapid heat diffusion as the layers are being consolidated can be reduced when a suitable print speed is chosen preventing defects between the layers. High printing speeds are described to decrease the cooling time of the filament which can influence the crystallization rate of the workpieces [35]. Additionally, the crystallinity of the polymer can be affected by annealing. PLA and PLA/ZrO_2_ were annealed and their mechanical properties were compared. Results suggested that the percentage of crystallinity was increased for the annealed filaments in comparison to the unannealed filaments. This was in accordance with the literature which described the influence of the heating and cooling process on the crystallization of the polymer [35].

In conclusion, the PLA/ZrO_2_ dental crown via the FDM 3D printing obtained an ultimate strength of 70.76 MPa. The mechanical properties for PLA materials fabricated with FDM technology such as tensile strength, tensile modulus and elongation are reported at a range of 15.5–72.2 MPa, 2.02–3.55 GPa and 0.5–9.2%, respectively [36]. Similarly, other authors have reported the elastic modulus of polyetheretherketone for dental restoration via FDM at 3–4 GPa [37]. The ASTM standard reported the minimum and maximum range for the ultimate tensile strength, elongation and compressive strength at 36–71.62 MPa, 3–20% and 41.26 MPa, respectively. It was reported that composite resins for posterior teeth should have a Young’s modulus equal to or higher than 18.5 MPa [38]. Our results obtained a Young’s modulus of 0.85 GPa after annealing. Although our results were within the typical ranges for FDM, the mechanical properties of human dentures—such as the compressive (384 MPa) and the fracture strength (molar = 305 MPa; premolar = 248 MPa)—was found to be over five-folds higher than the compressive strength of our workpieces. The compressive strength of conventional dental prosthetics using acrylic resin or nanohybrid composite resin via molding is reported around 85–360 MPa depending on the polymer material [39,40]. In terms of clinical applications, some limitations of our study suggest that our dental prothesis may only be suitable for short-term provisional restorations. However, the use of CAD/CAM technology is capable of providing same-day permanent dental restorations [5]. Therefore, further investigation on alternative filament compositions, porosity and other FDM parameters such as raster angle and print patterns are necessary to increase the strength of the workpieces [41,42]. The effect of the nozzle temperature and print bed can induce different types of porosity distribution patterns in the PLA structure, affecting the strength of the material [42]. Other researchers have reported higher fracture toughness of the PLA filament when printed at 45° in comparison to 90° via FDM [43].

## 5. Conclusions

A dental crown prototype was fabricated by FDM using PLA/ZrO_2_ synthetic resin with glycerol and a silane coupling agent as a binder. This study demonstrated the effectiveness of the synthetic resin to form strong complex shapes such as dental structure. High tensile strengths and high compressive strengths were achieved for the composites where the factors for filament extrusions and FDM printing parameters were optimized. The optimal condition for filament extrusion were melting temperature of 193 °C, PLA/zirconia ratio of 17 wt.% and screw speed of 25 rpm. The optimum ZrO_2_ ratio in the filament extrusion optimization achieved the highest tensile strength with consistent filament diameter size. The FDM printing parameter with the optimal compressive strength used a nozzle temperature of 210 °C and infill density at 100%. After 3D printing, the workpieces were annealed. The thermal and mechanical properties of the annealed composite resulted in increased heat resistance and compressive strength as compared to the unannealed composites. In view of this, results of the experiments in this study could be used as a guideline for the development of denture molding prototypes via 3D printing technology.

## Figures and Tables

**Table 1:** Experimental factors and levels for filament extrusion.

Factors	Level	
	Low (−)	High (+)
Melting temperature (°C)	195	205
PLA/ZrO_2_ ratio (wt.%)	10	20
Screw speed (rpm)	20	30

**Table 2:** Experimental factors and level for FDM 3D printing.

Factors	Level	
	Low (−)	High (+)
Nozzle temperature (°C)	190	210
Infill density (%)	80	100
Print speed (mm s^−1^)	20	100

**Table 3:** Tensile strength and DSC physical characteristics of different PLA filament compositions.

Sample	Ultimate Tensile Strength (MPa)	Elongation (%)	Young’s Modulus (GPa)	Yield Strength (MPa)	Glass Transition (°C)	Crystallization (°C)	Melting (°C)
PLA	22.68 ± 1.18 ^a^	14.55 ± 0.61 ^a^	1.14 ± 0.08 ^a^	15.08 ± 1.66 ^a^	62.56	93.32	175.38
PLA/ZrO_2_	14.42 ± 2.10 ^c^	10.17 ± 3.21 ^b^	1.05 ± 0.07 ^a^	10.40 ± 1.19 ^b^	61.22	85.83	170.58
PLA/ZrO_2_/glycerol	17.94 ± 0.79 ^b^	15.85 ± 2.11 ^a^	1.02 ± 0.07 ^a^	12.15 ± 0.68 ^b^	64.90	98.19	177.58
PLA/ZrO_2_/silane	21.09 ± 1.06 ^a^	13.69 ± 1.00 ^ab^	1.14 ± 0.09 ^a^	14.96 ± 2.04 ^a^	64.90	98.19	177.58
PLA/ZrO_2_/silane/glycerol	22.91 ± 0.99 ^a^	15.45 ± 1.69 ^a^	1.19 ± 0.15 ^a^	16.37 ± 1.82 ^a^	61.41	88.02	174.43

Values expressed as the mean ± SD were derived from the analysis of triplicate samples. Statistical analysis between the same column was performed by post hoc Tukey test with the significance level at *p* < 0.05, represented by lowercase superscript letters.

**Table 4:** Comparison of thermal and mechanical properties of annealed PLA and PLA/ZrO_2_ composites.

Sample	Cold Crystallization		Re- Crystallisation		Melting		Crystallinity *χ_c_* (%)
	T_cc_ (°C)	Δ*H_c_* (J g^−1^)	T (°C)	Δ*H_m°_* (J g^−1^)	T_m_ (°C)	Δ*H*_m_ (J g^−1^)	
PLA	89.5	3.82	156.5	1.14	175.2	8.08	3.74
Annealed PLA	*N/A	0.00	155.3	1.18	174.3	8.81	7.47
PLA/ZrO_2_	100.5	4.62	162.2	0.72	177.5	8.90	5.94
Annealed PLA/ZrO_2_	*N/A	0.00	159.3	1.20	177.0	10.63	9.43

Values expressed as the mean ± standard deviation were derived from the analysis of triplicate samples. Statistical analysis between the same column was performed by post hoc Tukey test with the significance level at *p* < 0.05. *N/A (no peaks were detected).

## Data Availability

Not applicable.

## Appendix A

Ultimate tensile strength is the stress at which a material breaks due to the load or force (*P*) divided by the original cross-sectional area (*a*), measured in Pascals [44].

(A1) $$Ultimate\;Tensile\;strength\;\left(MPa\right)=\frac{P}{a}$$

Elongation at break is the ratio between increased length (*L*) and initial length (Δ*L*) after breakage, measured in percentages [44].

(A2) $$Elongation\;at\;break\;\left(\%\right)=\left(\frac{\Delta L}{L}\right)\times 100\;$$

Young’s modulus is the ratio of the stress (*σ*) applied to the material along the longitudinal axis of the specimen tested and the strain (*ε*) measured on the same axis [44].

(A3) $$\sigma \;\left(stress\right)=\frac{F}{A}$$

(A4) $$\varepsilon \;\left(strain\right)=\frac{\Delta L}{L_{0}};\;\Delta L=L-L_{0}$$

(A5) $$Young’s\;Modulus\;\left(GPa\right)=\frac{\sigma }{\varepsilon }$$
